# Loss of ARID1A leads to a cold tumor phenotype via suppression of IFNγ signaling

**DOI:** 10.1038/s41598-025-91688-4

**Published:** 2025-03-13

**Authors:** Pamela M. Watson, Chelsea A. DeVaux, Kevin W. Freeman

**Affiliations:** 1 Department of Genetics, Genomics, and Informatics, University of Tennessee Health Science Center, Memphis, TN USA; 2 Center for Cancer Research, University of Tennessee Health Science Center, Memphis, TN USA

**Keywords:** Paediatric cancer, Cancer epigenetics, Tumour immunology

## Abstract

The collapse of inflammatory signaling that recruits cytotoxic immune cells to the tumor microenvironment contributes to the immunologically cold tumor phenotype in neuroblastoma (NB) and is a barrier to NB immunotherapy. Multiple studies have reported that *MYCN* amplification, a trait of high-risk NB, correlates with a loss of inflammatory signaling; but *MYCN* also correlates with 1p36 deletions in NB where the SWI/SNF chromatin remodeling complex subunit *ARID1A* (1p36.11) is located. ARID1A is known to support inflammatory signaling in adult cancers but its role in NB inflammatory signaling is unexplored. We find *MYCN* overexpression causes a stronger inflammatory response to interferon-gamma (IFNγ). ARID1A knockdown causes a weaker inflammatory response and reduces IFNγ induced gene signatures for the transcription factor interferon response factor 1 (*IRF1*). We found ARID1A is a functional interactor of IRF1 by co-immunoprecipitation studies, and ARID1A silencing causes loss of activating chromatin marks at the IRF1 target gene *CXCL10*. We model that IRF1 uses ARID1A containing SWI/SNF to promote CXCL10 in response to IFNγ. Our work clarifies that the loss of ARID1A, which tightly associates with *MYCN* amplification, causes reduced inflammatory signaling. This work finds that ARID1A is a critical regulator of inflammatory signaling in NB and provides rationale for testing immune therapies in *MYCN* amplified NB that are effective in adult ARID1A mutated cancers.

## Introduction

Neuroblastoma (NB) is a highly aggressive, heterogeneous pediatric cancer that arises from the sympathetic nervous system (SNS) and originates from neural crest cells (NCCs). NB is the most common extracranial cancer in pediatric patients and accounts for 15% of the total number of pediatric cancer deaths ^1,2^. This high mortality rate is the result of the metastatic, immune evasive, and treatment resistant characteristics of the disease ^1^. The success of anti-GD2 immunotherapy and the promise of other immunotherapies in NB has led to an increased focus on using immunotherapy to overcome the resistance of high-risk NB to standard of care treatments ^3–5^.

NB is considered an immunologically cold tumor, a designation characterized by a lack of tumor infiltrating CD8+ T cells and natural killer NK-T cells to the tumor microenvironment, low MHC-I expression, decreased amounts of antigen presentation, as well as an overall lack of tumor antigens due to its low mutational burden ^6^. Inflammatory responses are integral to the recruitment, differentiation, and activation of tumor infiltrating immune cells ^7,8^. In gene expression studies, NB has the lowest enrichment score for the Hallmark Inflammatory Response gene set of all cancer types sampled in gene set enrichment analysis (GSEA) ^9^. Poor inflammatory signaling could significantly contribute to NB’s cold tumor phenotype as it does in adult cancers like endometrial cancer, ovarian cancer, and melanoma ^10,11^.

High-risk NB is often characterized by *MYCN* gene amplification ^12,13^. *MYCN* has been implicated as a potential factor in the immune suppressive, or “cold” phenotype, in NB ^3,14^ as *MYCN* amplification correlates with low immune infiltrate signatures ^13^, lower amounts of NK-T cells and CD8+ T cells in tumors by mRNA expression ^15^, and lower amounts of MHC-I gene expression in tumors ^16^. By IHC, *MYCN* amplification also correlates to decreased immune infiltrate with marked differences in the amounts of CD8+ T cells and NK-T cells present in relation to *MYCN* WT tumors ^16,17^. However, *MYCN* amplification strongly correlates with 1p36 deletions in NB ^18,19^, raising the possibility that some of the immune characteristics attributed to N-MYC in NB may be due to loss of 1p36 genes.

The 1p36 loss of heterozygosity (LOH) occurs in around 29% of high-risk NB patients. We and others recently identified *ARID1A* as a 1p36 gene that is an N-MYC tumor suppressor ^20,21^. *ARID1A* is one of the highest mutated genes in many adult cancers including ovarian clear cell carcinoma, hepatocellular carcinoma, uterine carcinoma, as well as gastric, pancreatic, breast, colon, and lung cancer ^18,22^. ARID1A is a crucial part of epigenetic processes, determining areas of accessibility in the genome ^23,24^. ARID1A is part of the SWItch/Sucrose Non-Fermentable (SWI/SNF) complex, serving as the DNA binding domain of the complex BAF ^23^. This complex is responsible for the ejection or movement of nucleosomes along DNA, which allows for opening of chromatin for transcriptional machinery ^25–27^.

ARID1A has been implicated in shaping the immune phenotype in tumors as well as the efficacy of the response to immunotherapies in adult patients ^28–30^. A correlative study involving publicly available TCGA datasets demonstrated that ARID1A deficiency is a potential biomarker for immune checkpoint blockade success ^29^. Loss of ARID1A was shown to lead to a collapse of lymphocytic infiltrate by IHC stains of murine colon tumors ^28^ and human ovarian cancer samples ^29,30^. In ovarian clear cell carcinoma (OCCC) upon shRNA mediated knockdown of ARID1A, there was marked limited chromatin accessibility around interferon response factor I (IRF1) and IRF2 binding motifs leading to impaired gene expression and protein secretion of signaling chemokines CXCL9 and CXCL10 after interferon-gamma (IFNγ) treatment ^26^. These findings in the adult cancer field have informed our understanding of the role of ARID1A in tumor immunogenicity. Here, we identify that a core driver of the cold tumor phenotype in NB is ARID1A loss, and thus, we interrogate ARID1A dependent differences in immune signaling using human NB cells.

## Results

### *ARID1A* gene deletion status correlates with *MYCN* amplification and with a loss of cancer immune marker *CD8A* in patient tumors

**Fig. 1:**
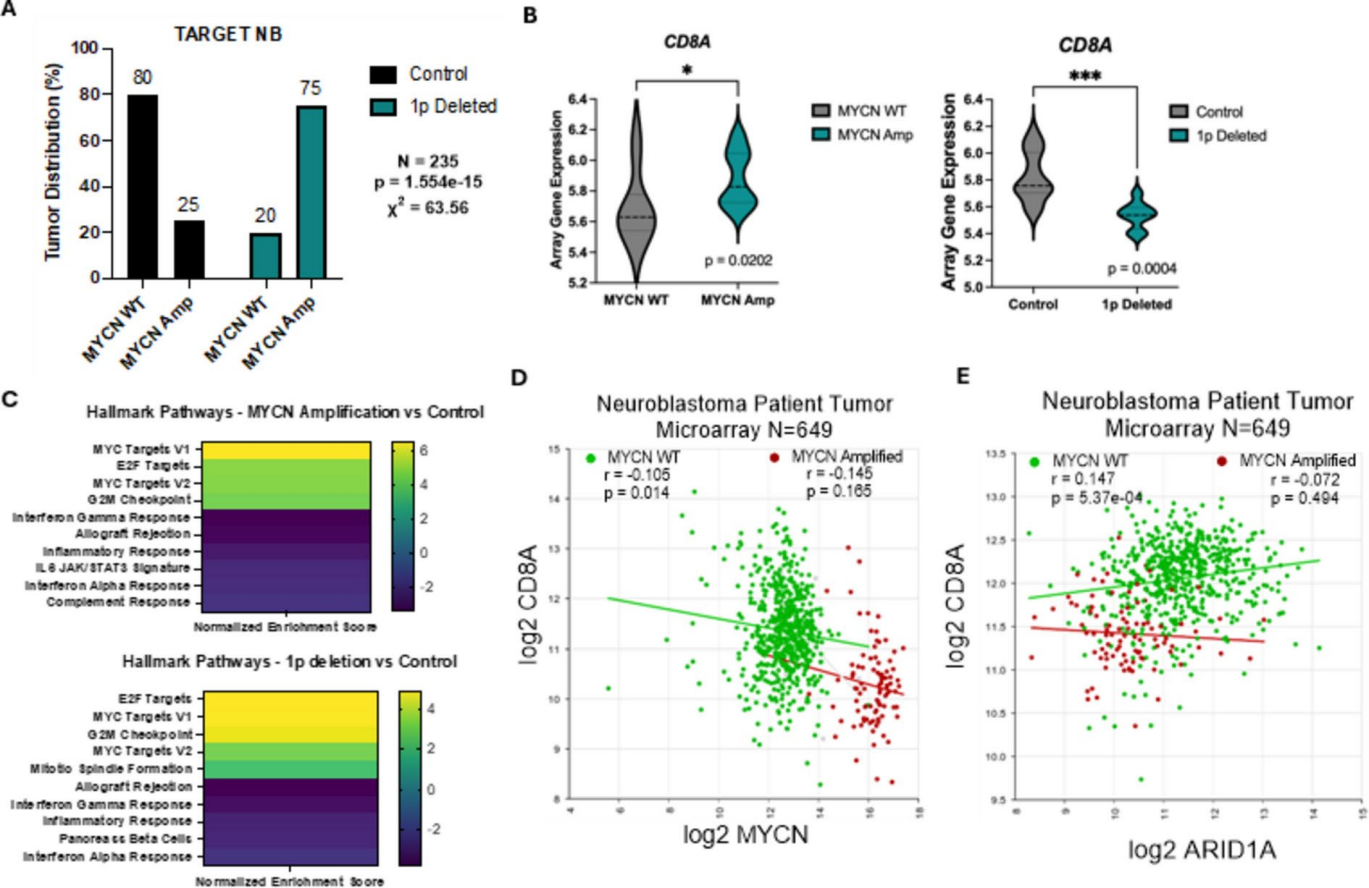
*ARID1A* gene deletion status correlates with *MYCN* amplification and with a loss of cancer immune marker *CD8A* in patient tumors. (**A–C**) Using the Xena Functional Genomics Explorer from UCSC, the TARGET Neuroblastoma dataset containing 717 patient samples was analyzed based on 1p status and *MYCN* amplification. (**A**) *MYCN* amplification correlates with 1p deletion in patient tumor samples (*p* = 1.554e-15) (n = 235). (**B**) In the *MYCN* amplified tumor samples, T cell marker (*CD8A*) mRNA levels increase compared to controls (*p* = 0.0202) (n = 28). In the 1p deleted tumor samples T cell marker (*CD8A*) mRNA levels drop significantly compared to controls (*p* = 0.0003805) (n = 28). (**C**) Top 10 significantly differentially expressed gene sets in the *MYCN* amplified and 1p deleted data set in Xena. These samples were analyzed using the Mayan Lab’s Appyter for blitzGSEA. Yellow and green denote gene sets that are upregulated in *MYCN* amplified and 1p deleted tumor samples, and blue and purple denote gene sets that are downregulated in the samples. (**D**) R2 analysis of the correlation of *MYCN* and CD8A gene expression in Neuroblastoma tumors in the Kocak data set N = 649 samples. These samples are separated by *MYCN* amplification status. *MYCN* WT samples are in green (r = -0.105, *p* = 0.014) and *MYCN* amplified patient samples are in red (r = -0.145, *p* = 0.165). (**E**) R2 analysis of the correlation of *ARID1A* and *CD8A* gene expression in Neuroblastoma tumors in the Kocak data set N = 649 samples. These samples are separated by *MYCN* amplification status. *MYCN* WT samples are in green (r = 0.147, *p* = 5.37e-04) and *MYCN* amplified patient samples are in red (r = -0.072, *p* = 0.494). (A-E) *p < 0.1; **p < 0.01, ***p < 0.001, ****p < 0.0001.

A correlation between *MYCN* amplification and a cold tumor phenotype has been reported in NB, but *MYCN* amplification also correlates with 1p36 deletion, which we confirmed using the UCSC Xena Database to investigate patient TARGET data from >700 patient samples (Fig. 1A) ^27^. ARID1A, within the commonly deleted 1p36 region in NB, has a role in initiating inflammatory signaling in adult cancers by opening areas of chromatin to enable the transcription of type I T-helper signaling genes. To explore the potential contribution of 1p36 deletion and ARID1A loss versus *MYCN* amplification to NB inflammatory signaling, we again used TARGET. We found a positive correlation between *MYCN* amplification and the CD8+ T cell marker *CD8A,* while demonstrating a negative correlation between 1p deletion and *CD8A* (Fig. 1B). To explore specific pathways selected between 1p deleted and *MYCN* amplified samples, we used the UCSC Xena platform with the Mayan Lab’s Appyter, blitzGSEA, to find the top 10 differentially expressed gene sets in the groups within Xena (Fig. 1C). Many of the datasets that were downregulated in both *MYCN* amplified and 1p deleted groups were specific for interferon and inflammatory response. These samples were not exclusive of one another, so specific effects were difficult to establish, which led us to explore other datasets. To investigate further, we expanded our inquiry with additional patient samples. Using a public NB dataset from the R2 Genomics Analysis and Visualization Platform containing 649 samples, we found that *MYCN* expression does weakly negatively correlate with *CD8A* gene expression levels when *MYCN* is WT (Fig. 1D), while *ARID1A* loss shows a highly significant correlation with a decrease in *CD8A* levels when *MYCN* is WT. (Fig. 1E). These findings led us to focus on the causal relationship between N-MYC, ARID1A, and inflammatory signaling in NB, specifically IFNγ signaling cytokines.

### *MYCN* overexpression leads to an increase in IFNγ signaling

**Fig. 2:**
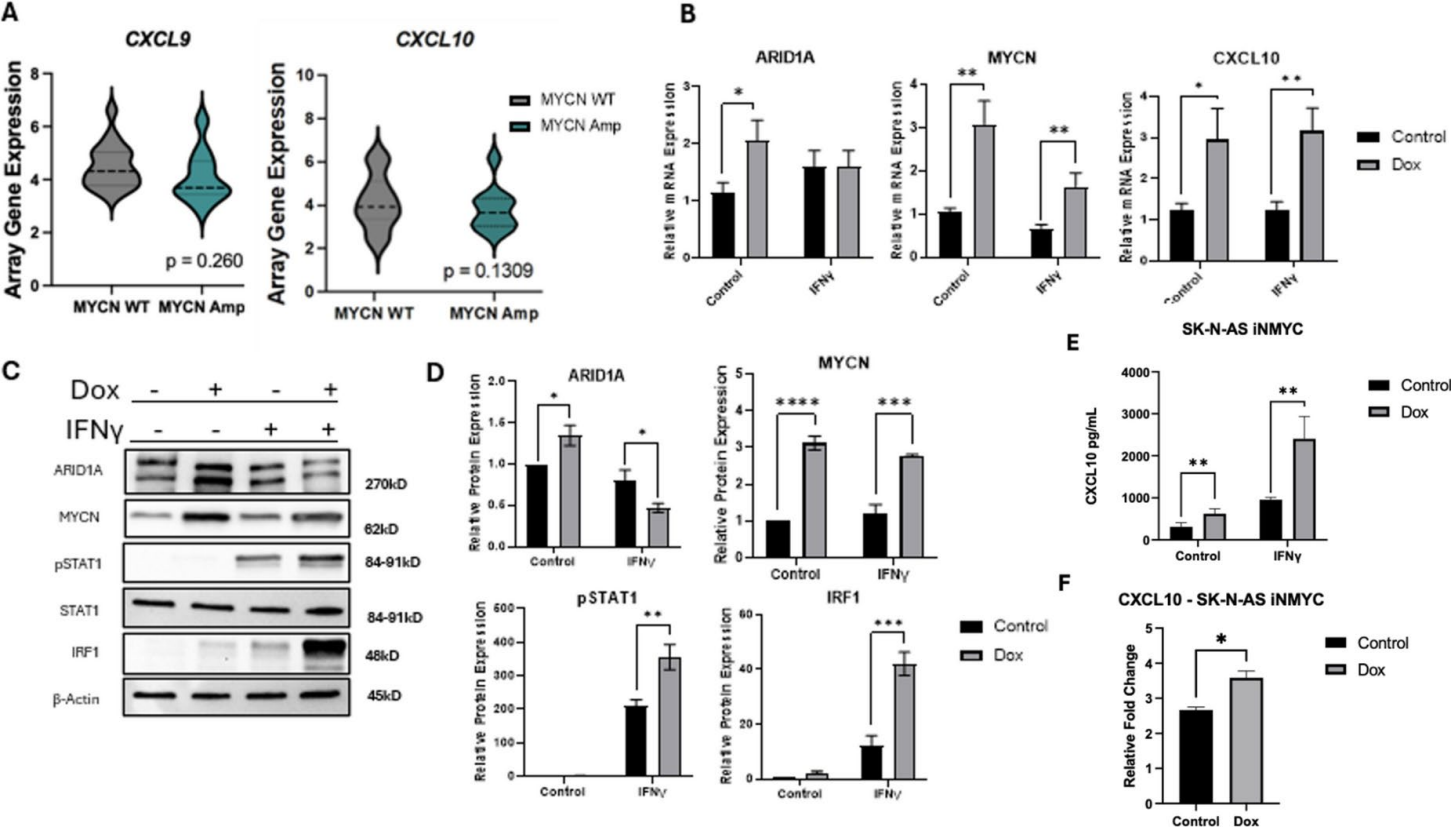
*MYCN* overexpression leads to an increase in IFNγ signaling. (**A**) IFNγ target chemokines, *CXCL9* and *CXCL10* mRNA expression levels decrease in *MYCN* amplified tumor samples in the UCSC Xena database (*p* = 0.2603) and (*p* = 0.1309) (N = 28). (**B**–**F**) A doxycycline inducible *MYCN* overexpression (OE) NB cell line was generated from SK-N-AS parental cells. Cells were treated with doxycycline for 72 h for *MYCN* induction. Cells were treated with IFNγ for 24 h prior to collection and subsequent analysis. (**B**) Relevant gene expression was quantified by real-time qPCR. Effect of *MYCN* OE and IFNγ treatment on SWI/SNF factor *ARID1A*, on *MYCN*, and on IFNγ target chemokine *CXCL10* (N = 3). (**C**) ARID1A, N-MYC, and relevant IFNγ signaling proteins pSTAT1, STAT1, and IRF1 were detected by Western blotting. One of 3 repeats is shown. (**D**) ARID1A, N-MYC, pSTAT1, and IRF1 protein expression was quantified (N = 3). (**E**) CXCL10 expression was quantified by ELISA (N = 3). (**F**) Relative fold change of CXCL10 protein levels between control and IFNγ treated samples of graph E. (A-F) *p < 0.1, **p < 0.01, ***p < 0.001, ****p < 0.0001.

Using the UCSC Xena Database to investigate patient TARGET data, we found positive relationships between *MYCN* amplification and increased immune suppression. Although not significant, there is a trend towards *MYCN* amplification being associated with decreased amounts of *CXCL9* and *CXCL10* gene expression in tumors (Fig. 2A). Before we investigated ARID1A loss and IFNγ signaling cytokines, we wanted to explore previous findings connecting *MYCN* amplification with the cold tumor phenotype. Previous studies found an in silico correlative relationship between *MYCN* and the NB cold tumor phenotype. ^14,15,17^. Silencing of N-MYC in NB did find in one study an increase in the inflammatory chemokine CCL2 which engages in the recruitment of NK cells ^27^. We wanted to investigate how enforced expression of N-MYC contributes to inflammatory signaling. We chose to stimulate cells with IFNγ to activate a signaling cascade leading to CXCL10 production. Using an SK-N-AS inducible *MYCN* overexpression cell line, which is *ARID1A* WT, we found that inducing *MYCN* leads to an increase in *ARID1A* and *MYCN,* and an increase in *CXCL10* gene expression with or without stimulus with IFNγ (Fig. 2B). Typically, *CXCL10*, an interferon inducible gene, requires interferon for expression but SK-N-AS had low basal expression. IRF1 protein expression was increased with *MYCN* overexpression, suggesting that greater N-MYC protein levels lead to an upregulation in multiple steps of IFNγ signaling (Fig. 2C,D) and its product chemokine CXCL10 (Fig. 2E,F). Additionally, we validated two other immune targets, *TAP1* and *GBP1*, and found they were increased as well (Supplemental 1). These findings demonstrate that increased N-MYC expression positively rather than negatively affects IFNγ specific cytokine signaling when ARID1A is wild-type.

### Knockdown of *ARID1A* leads to a decrease in *IRF1* and its downstream target chemokine *CXCL10*

**Fig. 3:**
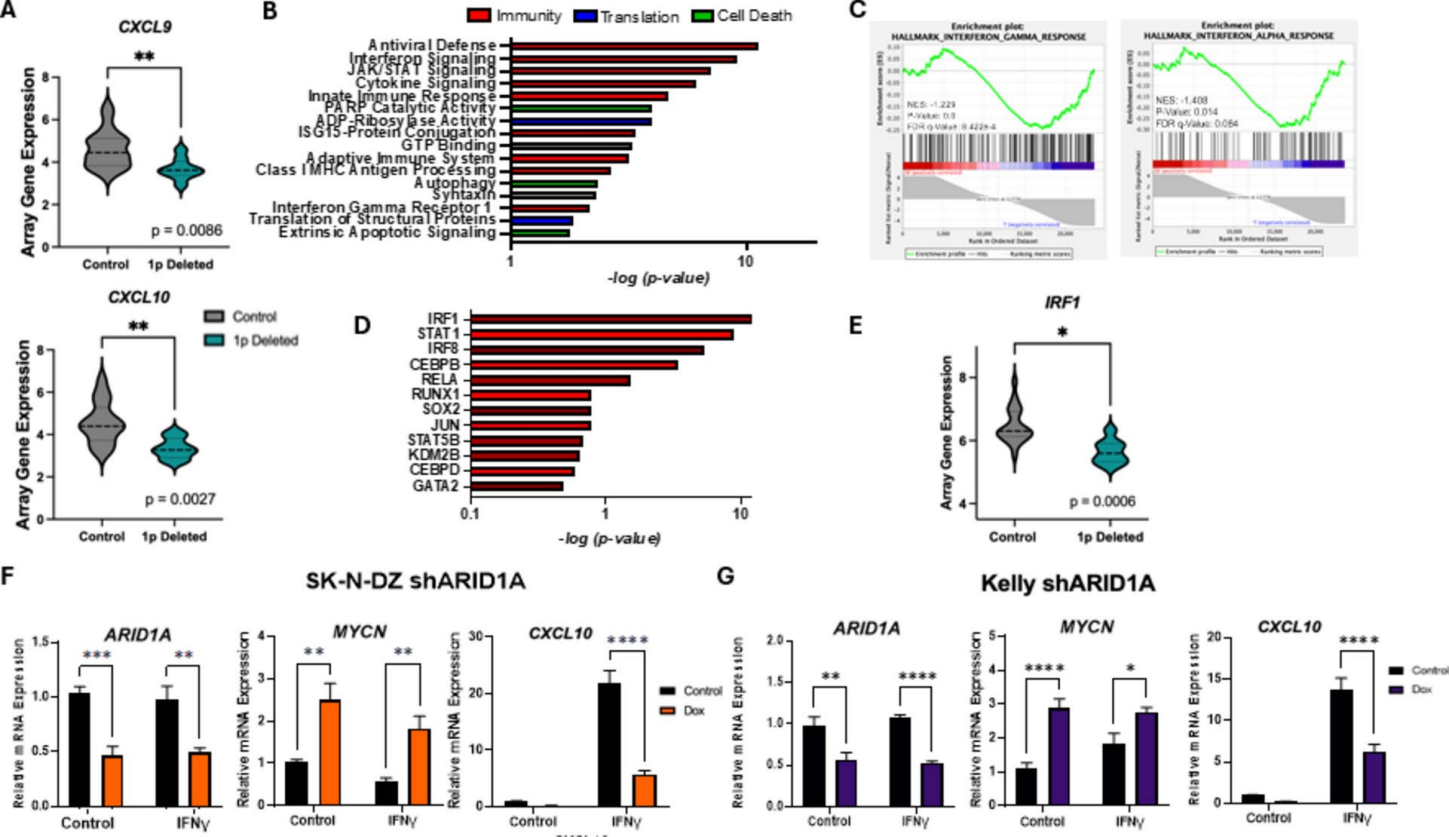
Knockdown of *ARID1A* leads to a decrease in IRF1 and its downstream target chemokine CXCL10. (**A**) IFNγ target chemokines, *CXCL9* and *CXCL10* mRNA expression levels are all significantly decreased in 1p deleted tumor samples in the UCSC Xena database (*p* = 0.0086) and (*p* = 0.0027) (N = 28). (**B**–**D**, **F**) Inducible *ARID1A*-knockout NB clones were generated from SK-N-DZ parental cells. Cells were treated with doxycycline for 72 h for shARID1A induction. Lines were treated with IFNγ for 24 h prior to collection and analysis. (**B**–**D**) RNAseq was conducted on SK-N-DZ shARID1A cells. (**B**) Effect of shARID1A knockdown on interferon signaling with IFNγ treatment. Differential expression between IFNγ treated (I) and shARID1A with IFNγ treatment (DI) was entered for DAVID pathway analysis (N = 6). (**C**) Gene set enrichment analysis (GSEA) was completed between I and DI groups for Hallmark Interferon Gamma Response (*p* = 0.0) and Hallmark Interferon Alpha Response (*p* = 0.014) (N = 6). (**D**) Top 12 differentially expressed immune related genes lost in the DI group compared to the I group in analysis by Enrichr for the CHEA Transcription Factor Targets dataset. (**E**) *IRF1* mRNA expression is significantly decreased in 1p deleted tumor samples in the UCSC Xena database (*p* = 0.0006) (N = 28). (**F**) Relevant gene expression was quantified by real-time qPCR. Effect of *ARID1A* knockdown and IFNγ treatment on SWI/SNF factor *ARID1A*, on *MYCN*, and on IFNγ target chemokine *CXCL10* (N = 3). (**G**) Inducible *ARID1A*-knockout NB clones were generated from Kelly parental cells. Cells were treated with doxycycline for 72 h for shARID1A induction. Lines were treated with IFNγ for 24 h prior to collection and analysis. Relevant gene expression was quantified by real-time qPCR. Effect of *ARID1A* knockdown and IFNγ treatment on SWI/SNF factor *ARID1A*, on *MYCN*, and on IFNγ target chemokine *CXCL10* (N = 3). (**A**–**G**) *p < 0.1; **p < 0.01, ***p < 0.001, ****p < 0.0001.​

Using the UCSC Xena Database to investigate patient TARGET data, we found positive relationships between 1p deletion and increased immune suppression regardless of *MYCN* amplification status, with significantly decreased amounts of *CXCL9* and *CXCL10* gene expression in tumors (Fig. 3A). Since ARID1A has a known relationship with tumor immunity in adult cancers, we interrogated the effects of ARID1A loss on cytokine signaling in NB. An inducible shARID1A knockdown clonal cell line was generated from the *MYCN* amplified NB cell line SK-N-DZ, which is unique as it is *MYCN* amplified as well as wildtype for *ARID1A* ^28^. We conducted RNA sequencing after doxycycline treatment and IFNγ stimulation. The significant differentially expressed genes were entered for DAVID pathway analysis, which resulted in responses involved in immunity, protein translation, and cellular death (Fig. 3B). Gene set enrichment analysis (GSEA) was completed for these samples, and the Hallmark pathways for Interferon Gamma Response and Interferon Alpha Response were significantly enriched in the IFNγ treated group with WT *ARID1A* (Fig. 3C). Within this group, the most significant differentially expressed genes were entered into Enrichr, which produced a table of associated transcription factors, the most significant of which is *IRF1* (Fig. 3D). We then investigated *IRF1* gene expression levels when 1p was deleted in NB tumor samples in Xena. When deleted, there is a significant loss of *IRF1* expression in patient data (Fig. 3E). This led us to explore more specifically the effects of ARID1A loss on IRF1 activity in the JAK/STAT pathway. To do this, we chose to stimulate cells with IFNγ to activate a signaling cascade leading to CXCL10 production. The loss of *ARID1A* in the SK-N-DZ knockdown line led to an increase in *MYCN* gene expression and a decrease in *CXCL10* gene expression upon IFNγ stimulation (Fig. 3F and Supplemental Fig. 1A). Another inducible shARID1A knockdown clonal cell line was generated from the *MYCN* amplified NB Kelly cell line. Kelly reflects the majority of *MYCN* amplified NB which has a one chromosomal deletion of the 1p36 region creating a cell line that has a loss of heterozygosity (LOH) for *ARID1A* ^27^. This deeper knockdown allowed us to examine to what extent the degree of loss in *ARID1A* correlates best with the immune suppressive phenotype. As before, when *ARID1A* was knocked down, *MYCN* was increased and *CXCL10* gene expression was decreased upon IFNγ stimulation (Fig. 3G and Supplemental Fig. 1B). Additionally, we validated two other IFNγ inducible immune targets, *TAP1,*which promotes major histocompatibility (MHC) class I antigen presentation and *GBP1*,which supports innate immunity and found they were decreased upon ARID1A knockdown after IFNγ stimulation (Supplemental 4).

### Knockdown of ARID1A leads to a decrease in IFNγ signaling in NB cells

**Fig. 4:**
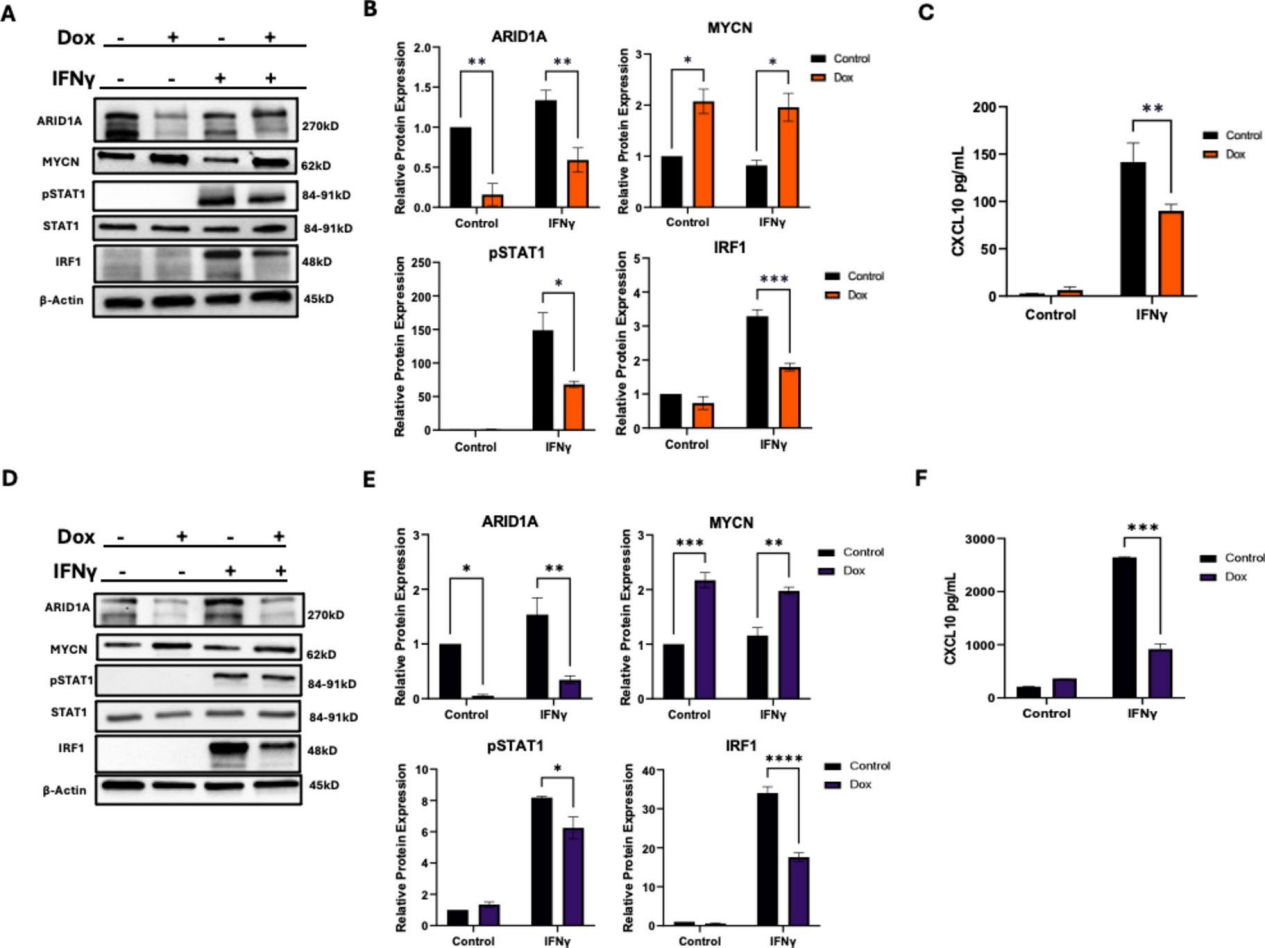
Knockdown (KD) of *ARID1A* leads to a decrease IFNγ signaling in NB cells. (**A**–**C**) Inducible *ARID1A*-knockout NB clones were generated from SK-N-DZ parental cells. KD lines were treated with doxycycline for 72 h for shARID1A induction. Lines were treated with IFNγ for 24 h prior to collection and analysis. (**A**) ARID1A, N-MYC, and relevant IFNγ signaling proteins pSTAT1, STAT1, and IRF1 were detected by Western blotting in SK-N-DZ cells. One of 3 repeats is shown. (**B**) ARID1A, MYCN, pSTAT1, and IRF1 protein expression was quantified and graphed (N = 3). (**C**) Chemokine expression of CXCL10 was quantified by ELISA in SK-N-DZ cells (N = 3). (**D**–**F**) Inducible ARID1A-knockout NB clones were generated from Kelly parental cells. KD lines were treated with doxycycline for 72 h for shARID1A induction. Lines were treated with IFNγ for 24 h prior to collection and analysis. (**D**) ARID1A, MYCN, and relevant IFNγ signaling proteins pSTAT1, STAT1, and IRF1 were detected by Western blotting in Kelly cells. One of 3 repeats is shown. (**E**) ARID1A, MYCN, pSTAT1, and IRF1 was protein expression was quantified and graphed (N = 3). (**F**) Chemokine expression of CXCL10 was quantified by ELISA in Kelly (N = 3). (A-F) *p < 0.1; **p < 0.01, ***p < 0.001, ****p < 0.0001.​

Using both shARID1A cell lines, we interrogated the components of the IFNγ signaling pathway. To do this, we chose to stimulate cells with IFNγ to activate a signaling cascade leading to CXCL10 production. Decreased ARID1A protein levels led to a decrease in pSTAT1 and IRF1 by Western Blot (Fig. 4A and B, and Supplemental Fig. 2A and 2B) and CXCL10 by ELISA upon IFNγ stimulation (Fig. 4C and Supplemental Fig. 2C). A deeper knockdown of ARID1A protein levels also led to a decrease in pSTAT1 and IRF1 in Kelly cells by Western Blot (Fig. 4D,E, and Supplemental Fig. 2D,E) and CXCL10 by ELISA upon IFNγ stimulation (Fig. 4F and Supplemental Fig. 2F). In particular, the ELISA data found that a more pronounced decrease in ARID1A leads to more dramatic immune loss by cytokine expression. In total, this supported our initial findings seen in the Xena and TARGET tumor samples but also provided key insight into which areas of the IFNγ signaling pathway can change upon ARID1A loss.

### Knockdown of *ARID1A* leads to a decrease in activating histone marks at *IRF1* and *CXCL10* promoters and enhancers

**Fig. 5:**
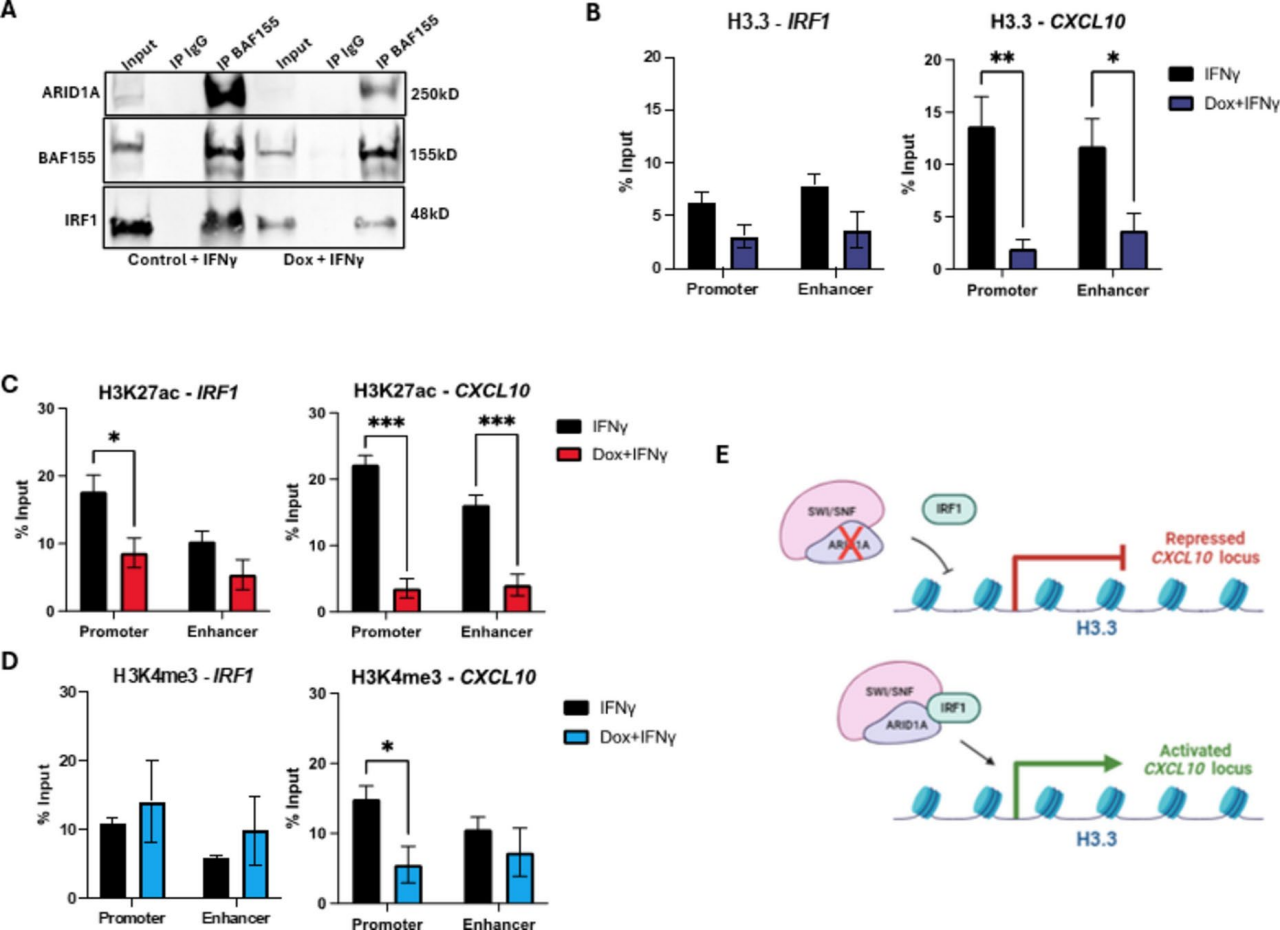
Knockdown of *ARID1A* leads to a decrease in activating histone marks at *IRF1* and *CXCL10* promoters and enhancers. (**A**–**D**) Inducible *ARID1A*-knockout NB clones were generated from Kelly parental cells. KD lines were treated with doxycycline for 72 h for shARID1A induction. Lines were treated with IFNγ for 24 h prior to collection and analysis. (**A**) Immunoprecipitation-Western blot (IP-WB) was performed on shARID1A Kelly cells treated with IFNγ for 24 h prior to collection and analysis. BAF155 (SMARCC1) was used for the IP pull down, and IRF1 was shown by WB. One of 3 blots is shown (N = 3). (**B**–**D**) ChIP-qPCR was completed using shARID1A Kelly cells treated with IFNγ for 24 h prior to collection and analysis. Immunoprecipitation using antibodies for (**B**) H3.3, (**C**) H3K27ac, and (**D**) H3K4me3 yielded genomic material for subsequent qPCR for promoter and enhancer sequences for *IRF1* and *CXCL10*. Results are shown as enrichment relative to percent of total input before IP. (**E**) Predicted model of the interaction between ARID1A and IRF1, and the affects of ARID1A loss on downstream *CXCL10* transcription and signaling. (**B**–**D**) *p < 0.1; **p < 0.01, ***p < 0.001, ****p < 0.0001.

ARID1A was previously reported to interact with IRF3, a paralog of IRF1, in HEK293T cells; but it was not determined if the IRF3 interaction was specific to ARID1A or through a different subunit of the SWI/SNF complex ^30^. Based on the significant effects on IRF1 targets genes that we observed when *ARID1A* is silenced (Fig. 3D), we wanted to determine if IRF1 also interacts with SWI/SNF and if that interaction is ARID1A dependent. To investigate this, the Kelly shARID1A cell line, which has the more potent *ARID1A* knockdown, was treated with doxycycline for 72 h and stimulated with IFNγ for 24 h. BAF155 (SMARCC1), a core subunit of SWI/SNF complexes, was immunoprecipitated and visualized by Western blot along with ARID1A and IRF1. Overall protein expression of BAF155 was equivalent between the treatment groups, whereas IRF1 expression decreased with decreased ARID1A expression after doxycycline induced knockdown (Fig. 5A). Our findings indicate that IRF1 interacts with SWI/SNF complexes. However, the interaction is not ARID1A specific since the degree of loss of IRF1 pulldown seems to be mostly driven by loss of IRF1 expression as the loss between the input lanes are similar to the loss between the IP-BAF155 lanes (Fig. 5A). This data helps us understand the impact of ARID1A loss on IRF1 function in IFNγ signaling, specifically that the effects of downstream transcriptional loss can be compounding leading to decreased universal loss in the pathway.

ARID1A can alter histone marks associated with chromosome accessibility at regulatory elements to affect gene expression ^31,32^ H3.3 is enriched at active and highly accessible regulatory elements ^33^, with ARID1A having a critical role in maintaining H3.3 at these sites ^34^. Loss of ARID1A causes loss of the activating H3K27ac mark at regulatory elements ^35^. ARID1A was also recently shown to 1. Interact with IRF3 in macrophages, 2. Increase accessibility of IRF3 to its targets, and 3. Recruit the histone methyltransferase NSD2 to methylate H3K4 ^36^. We therefore wanted to assess the effects of ARID1A silencing on histone marks known to be regulated by ARID1A at the gene regulatory sites for *IRF1* and its target gene *CXCL10*. To do this, we performed chromatin immunoprecipitation quantitative PCR (ChIP-qPCR) using the Kelly shARID1A cell line. Again, Kelly cells were treated with doxycycline for 72 h and stimulated with IFNγ for 24 h. ChIP was performed using H3.3, H3K27ac, or H3K4me3 antibodies for immunoprecipitation pulldown after chromatin fixation and shearing (Fig. 5B–D). Afterwards, *IRF1* and *CXCL10* promoter and enhancer sequences were examined by qPCR. We observe marked and significant decreases in histones and histone marks in the doxycycline treated groups for *CXCL10* promoters and enhancers, except for the H3K4me3 mark at the *CXCL10* enhancer (Fig. 5B–D). The only significant change for *IRF1* in response to silencing of *ARID1A* was H3K27ac at the *IRF1* promoter; suggesting ARID1A is not directly acting on the *IRF1* regulatory loci, but instead indirectly affecting *IRF1* expression through some other mechanism (Fig. 5C). The impact of *ARID1A* silencing on IRF1 target genes (Fig. 3B), its interaction with IRF1 (Fig. 5A), and its roles in maintaining activating histone marks at the IRF1 target gene *CXCL10* (Fig. 5B–D) leads us to model that IRF1 recruits ARID1A containing SWI/SNF complexes to maintain chromatin accessibility and to increase activating chromosome marks at IRF1 target genes (Fig. 5E).

## Discussion

In this study, we found a link between ARID1A and IRF1 in IFNγ specific inflammatory signaling in NB. This link could potentiate a greater understanding of the cold tumor phenotype in NB and open opportunities to increase the success of immunotherapy in the future. For patients with NB, specifically high-risk NB, treatment options are extremely limited. Immunotherapy in NB has seen success with anti-GD2 therapy ^5^, but the cold tumor phenotype of NB is a significant barrier to 1. improving anti-GD2 therapy as many patients show no or limited response, and 2. improving other immunotherapeutic approaches such as checkpoint inhibitors ^35^. One proposed reason for immunotherapy failure is the limited number of cytotoxic cells within the tumor microenvironment ^3–5^. A key component of cytotoxic T cell and NK cell recruitment is the initiation of inflammatory signaling from the tumor to the immune system ^7^. In adult cancers, this relationship is well known and studied ^17–19^, but there is limited understanding within pediatric cancers ^37^. *MYCN* amplification has been implicated in many studies about the cold tumor phenotype in NB. However, these studies are mostly correlative, in silico findings from the TARGET or PECAN databases ^11–13^. Since *MYCN* amplification also strongly correlates with 1p36 loss in NB tumors, 1p36 genes could also be contributing to the cold tumor phenotype. When we looked at datasets involving both *MYCN* amplification and 1p deletion, we noted that the most robust *CD8A,* a marker for cytotoxic T-cells, gene expression changes were seen in 1p deleted samples when *MYCN* was WT. We also observed that 1p deletion associated with the same downregulated inflammatory signaling pathways that previously groups had correlated with *MYCN* amplification, thus indicating that a 1p36 genes might be responsible for this phenotype.

We focused on IFNγ specific inflammatory signaling as it was the pathway we observed that most associated with *MYCN* amplification and 1p36 loss. We found that the greatest effects on cytokine collapse after IFNγ stimulation were when *ARID1A,* a 1p36 gene known to regulate inflammatory signaling, was knocked down rather than with *MYCN* overexpression indicating ARID1A as the more relevant regulator of IFNγ specific inflammatory signaling in NB. Enforced N-MYC overexpression instead led to increased IFNγ signaling. This suggests the association between *MYCN* amplification and immune suppression reported in the literature is instead due to the association of *MYCN* amplification with *ARID1A* loss and ARID1A’s critical role in IFNγ signaling.

IRF1 is a transcription factor found in the IFNγ signaling pathway ^7,25^. Upon stimulation, IRF1 binds to DNA allowing for IFNγ regulated transcription to take place. *CXCL10* is a direct transcriptional target of IRF1. Here, we propose that IRF1 recruits ARID1A to control transcription of inflammatory cytokines by opening areas of chromatin. We observe that loss of *ARID1A* leads to significant decreases in histone marks associated with chromosome accessibility at *CXCL10* providing a mechanistic explanation for its loss of expression. Although ARID1A itself is not druggable, inhibiting opponents of SWI/SNF could present new therapeutic opportunities when paired with immunotherapies. In adult cancers, inhibiting EZH2, a component of PRC2, has been successful in restoring immunogenicity in adult cancers with *ARID1A* mutations ^38,39^. Our findings indicate that loss of *ARID1A*, rather than *MYCN* amplification, might be the better biomarker for responsiveness to EZH2 therapies in combination with immunotherapies in NB.

## Materials and Methods

### Analysis of the TARGET Neuroblastoma dataset

The TARGET Neuroblastoma (N = 717) dataset was accessed, and correlations between the 1p-deleted group and the *MYCN* amplified group were gathered using Xena database from UCSC (https://xenabrowser.net/). We transformed each expression value using the Log2 function, and each graph was plotted by subsection. We assessed the significant difference using the unpaired Wilcoxon crossover test. The top ten differentially expressed genes were found using the blitzGSEA application from the Mayan Lab (https://appyters.maayanlab.cloud/#/). Significance was measured as *p* < 0.05.

### R2 genomic analysis

The Kocak Neuroblastoma tumor (N = 649) dataset was accessed through the R2 Genomics Analysis and Visualization Platform (http://r2.amc.nl). mRNA expression levels were measured by correlations of two specific genes at a time. Samples were divided between two groups: *MYCN* amplified in red and Non-*MYCN* amplified in green. Linear best fit is shown with a line. Statistics were generated using the Pearson’s Product Moment Correlation Coefficient, generating an “r” value and a “*p*” value. Significance was measured as *p* < 0.05.

### Generation of *MYCN* overexpressing cells

SK-N-AS cells (ATCC, CRL-2137) were first transduced with regulatory plasmid pcDNA6/TR, a CMV driven Tet-Repressor construct (Invitrogen, K1020-1) and then stably selected with blastocydin to establish SK-N-AS cells with Tet-repressor. These SK-N-AS Tet-repressor cells were then transduced into a plasmid, pcDNA4/TO plus containing two Tet operons downstream of a CMV promoter and with doxycycline inducible N-Myc (iN-Myc-AS) to generate pooled SK-N-AS cells that overexpressed *MYCN* when treated with doxycycline.

### Generation of shARID1A cells

shRNA clonal lines were generated from Kelly (Millipore Sigma, 92,110,411) and SK-N-DZ (ATCC, CRL-2149) parental cell lines. The shRNA sequences were designed according to the TRC1 library (Sigma-Aldrich, TRCN0000358749, TRCN0000344709, TRCN0000059091, and TRCN0000059090, referred to in the manuscript as sh1, sh2, sh4, and sh5, respectively) targeting *ARID1A*. The RNAs were cloned into the lentiviral vector Tet-pLKO-puro (Plasmid #21,915) and lentivirus for each shRNA was generated with 293 T cells using second-generation lentiviral system. This plasmid construct was packaged into the cells using lentiviral transduction. Puromycin (Millipore Sigma, P9620) was added to select for transduced cells. To establish clones, cells were seeded at low density and treated with puromycin for 5 days to a month. Clonal lines were picked using cloning rings and cultured separately than pooled cells. Clones showing the most robust ARID1A knockdown were shown in the main manuscript. sh1 and sh2 were most effective in Kelly cells, and sh4 and sh5 were most effective in SK-N-DZ cells. Secondary clone data is provided in the supplemental figures.

### NB cell treatment time course

Neuroblastoma cells from Kelly and SK-N-DZ clones were cultured under puromycin (1µg/mL) selection one week prior to treatment (Millipore Sigma, P9620). Cells were passaged the night before doxycycline treatment at a density of 100,000 cells in a 10cm dish. Cells were treated with doxycycline (1µg/mL) (Millipore Sigma, D3447) at day one and day three to knock down ARID1A , and eventually treated with human recombinant IFNγ (50µg/mL) (Peprotech, 300–02) on day four for 24 h to induce inflammatory signaling. Samples were collected at the 24-h mark on day five.

### Total RNA extraction

Total RNA of the NB cell lines was extracted using the RNeasy Mini Kit [QIAGEN]. The extracted RNA was then reverse transcribed into cDNA using the High-Capacity cDNA reverse transcription kit (Thermo Fisher Scientific).

### RT-qPCR analysis

mRNA expression levels within the cell lines were analyzed by qPCR using TaqMan Fast advanced master mix (Thermo Fisher Scientific) and QuantStudio 3 Real-Time PCR system (Thermo Fisher Scientific). Specific probes for the genes investigated in this study include: *ARID1A* (assay ID, Hs00195664_m1), *MYCN* (assay ID, Hs00232074_m1), *EZH2* (assay ID, Hs00544830_m1), and *CXCL10* (assay ID, Hs00171042_m1) (all Thermo Fisher Scientific). Gene expression levels were calculated using the ΔΔCT method using Excel. Two housekeeping genes were used as internal controls: *PPIB* (assay ID, Hs00168719_m1) and *HPRT1* (assay ID, Hs02800695_m1). Each sample was run and analyzed in triplicate. Sample averages and standard errors were plotted using PRISM, and a two-way ANOVA was used to establish P-value.

### Western blot analysis

Protein expression levels within the cell lines were analyzed by Western blot using the iBright CL1500 imaging system (Thermo Fisher Scientific). Samples were run on identical gels in tandem and were cut before transfer and addition of antibodies. Specific antibodies (Cell Signaling) for proteins investigated in this study include: ARID1A (D2A8U), pSTAT1 (Tyr701, 58D6), STAT1 (D1K9Y), N-MYC (D4B2Y), EZH2 (D2C9), IRF1 (D5E4), and β-ACTIN (8H10D10). Secondary antibodies include rabbit (7074) and mouse (7076). Blots were quantified using Image J software (https://imagej.net/ij/). Sample averages and standard errors were plotted using PRISM, and multiple T-tests were used to establish P-value.

### ELISA

Protein expression of the chemokine CXCL10 was measured by ELISA using the IP-10 (CXCL10) Human ELISA Development Kit (ABTS) (Peprotech, Thermo Fisher Scientific, 900-K39). Results were visualized using the 1-Step ABTS substrate solution (Thermo Fisher Scientific, 37,615) and the Biotek microplate reader (Agilent). Sample averages and standard errors were plotted using PRISM, and multiple T-tests were used to establish P-value.

### RNAseq

Total RNA of the NB cell lines was extracted using the RNeasy Mini Kit (QIAGEN). Samples were collected and analyzed by the University of Tennessee Health Science Center’s Molecular Resource Center to ensure quality control using the Agilent 2100 Bioanalyzer instrument. Samples were then sent to Novogene (USA) for sequencing using the Illumina NovaSeq 6000. Bioinformatic analysis was completed using R Studio. All sequencing data can be found on the Gene Expression Omnibus (https://www.ncbi.nlm.nih.gov/geo/) using accession GSE277050.

### Pathway analysis

Gene-set enrichment analysis was completed using the GSEA software (https://www.gsea-msigdb.org/gsea/index.jsp). Enrichr (https://maayanlab.cloud/Enrichr/) was used to determine pathways enriched in the RNAseq data. This analysis was performed on the top 200 genes of the sorted gene list based on the adjusted *p* value score.

### IP-WB

Immunoprecipitation of IgG (Cell Signaling, 7074S), BAF155 (Santa Cruz, sc-32763), and IRF1 (Santa Cruz, sc-514544) was executed using Pierce IP Lysis Buffer (ThermoFisher, 87,788) and Pierce Protein A/G Magnetic Beads (ThermoFisher, 88,802). Western blots were completed with antibodies (Cell Signaling) for BAF155 (D7F8S), ARID1A (D2A8U), ARID1B (E9J4T), ARID2 (D8D8U), and IRF1 (D5E4). Secondary antibodies (Cell Signaling) include rabbit (7074) and mouse (7076).

### ChIP-qPCR

ChIP was performed on Kelly cells using the ChIP-IT Express Kit (Active Motif, 53,008). Chromatin was sheared using the Bioruptor Pico instrument (Hologic Diagenode) and immunoprecipitation was conducted using IgG (Cell Signaling, 7074S), H3.3 (Millipore, 09–838), H3K27ac (Cell Signaling, 8173S), and H3K4me3 (Cell Signaling, 9751S). qPCR was conducted with custom probes for *IRF1* and *CXCL10* using the Custom TaqMan Assay Design Tool from ThermoFisher. Each sample was run using TaqMan Fast advanced master mix (Thermo Fisher Scientific) and QuantStudio 3 Real-Time PCR system (Thermo Fisher Scientific) and analyzed in triplicate. Sample averages and standard errors were plotted using PRISM, and a two-way ANOVA was used to establish P-value.

### Graphing and statistics

All graphs and statistics were built using PRISM software.

## Supplementary Information

Supplementary Information 1.

## Data Availability

All sequencing data can be found on the Gene Expression Omnibus (https://www.ncbi.nlm.nih.gov/geo/) using accession GSE277050.
