# Supplementary material for: Loss of ARID1A leads to a cold tumor phenotype via suppression of IFNγ signaling
Source: Sci Rep. 2025 Mar 13;15:8716. doi: 10.1038/s41598-025-91688-4 (PMC11906763; doi:10.1038/s41598-025-91688-4)
Supplement: Supplementary file 1 — Supplementary Information 1. [file 41598_2025_91688_MOESM1_ESM.docx]

**Supplemental Figures**

**
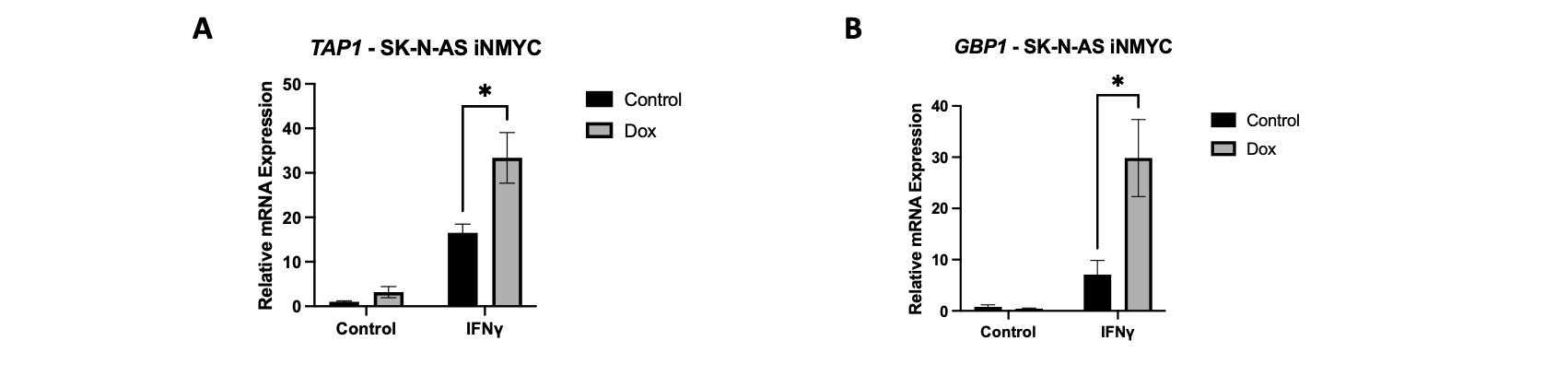
**

**Supplemental Figure 1: SK-N-AS with MYCN overexpression increases the gene expression of immune related genes.**

**(A-B)** SK-N-AS MYCN OE cells were treated with doxycycline for 72 hours for *MYCN* induction. Cells were treated with IFNγ for 24 hours prior to collection and subsequent analysis. Gene expression was measured by qPCR for antigen processing gene, **(A)** *TAP1*, and another interferon responsive gene, **(B)** *GBP1*. **(A-B)** *p < 0.1; **p < 0.01, ***p < 0.001, ****p < 0.0001.

**
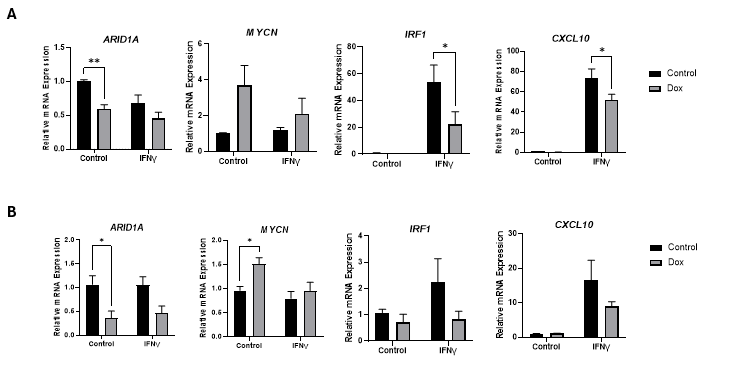
**

**Supplemental Figure 2: Knockdown of *ARID1A* leads to a decrease in chemokine *CXCL10* by mRNA and a decrease in overall IFNγ signaling by protein in additional SK-N-DZ and Kelly clones.**
**(A-B)** Inducible ARID1A-knockout NB clones were generated from SK-N-DZ parental cells. KD lines were treated with doxycycline for 72 hours for shARID1A induction. Lines were treated with IFNγ for 24 hours prior to collection and analysis. **(A)** Relevant gene expression in SK-N-DZ cells was quantified by real-time qPCR. Effect of ARID1A knockdown and IFNγ treatment on SWI/SNF factor *ARID1A*, on *MYCN*, and on IFNγ target chemokine *CXCL10* (N=3). **(B)** Relevant gene expression in Kelly cells was quantified by real-time qPCR. Effect of ARID1A knockdown and IFNγ treatment on SWI/SNF factor *ARID1A*, on *MYCN*, and on IFNγ target chemokine *CXCL10* (N=3). **(A-B)** *p < 0.1; **p < 0.01, ***p < 0.001, ****p < 0.0001.


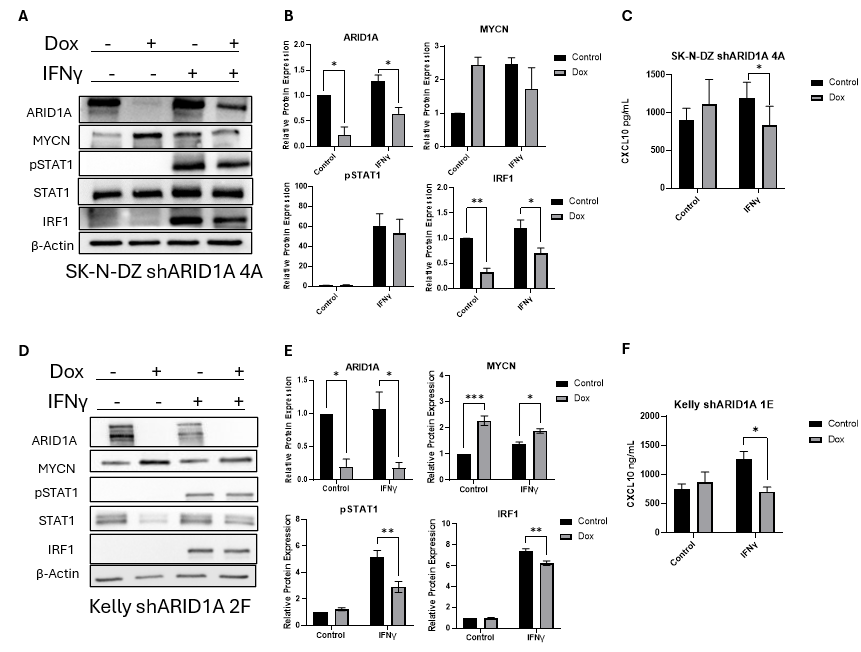


**Supplemental Figure 3: Knockdown of ARID1A leads to a dose dependent decrease IFNγ signaling in additional SK-N-DZ and Kelly clones.**
(**A-F)** Inducible ARID1A-knockout NB clones were generated from SK-N-DZ and Kelly parental cells. KD lines were treated with doxycycline for 72 hours for shARID1A induction. Lines were treated with IFNγ for 24 hours prior to collection and analysis. **(A)** ARID1A, MYCN, and relevant IFNγ signaling proteins pSTAT1, and IRF1 were detected by Western blotting in SK-N-DZ cells. One of 3 repeats is shown. **(B)** ARID1A, MYCN, pSTAT1, and IRF1 was protein expression was quantified and graphed (N=3). **(C)** Chemokine expression of CXCL10 was quantified by ELISA in SK-N-DZ cells (N=3). **(D)** ARID1A, MYCN, and relevant IFNγ signaling proteins pSTAT1, and IRF1 were detected by Western blotting in SK-N-DZ cells. One of 3 repeats is shown. **(E)** ARID1A, MYCN, pSTAT1, and IRF1 was protein expression was quantified and graphed (N=3). **(F)** Chemokine expression of CXCL10 was quantified by ELISA in SK-N-DZ cells (N=3). **(A-F)** *p < 0.1; **p < 0.01, ***p < 0.001, ****p < 0.0001.


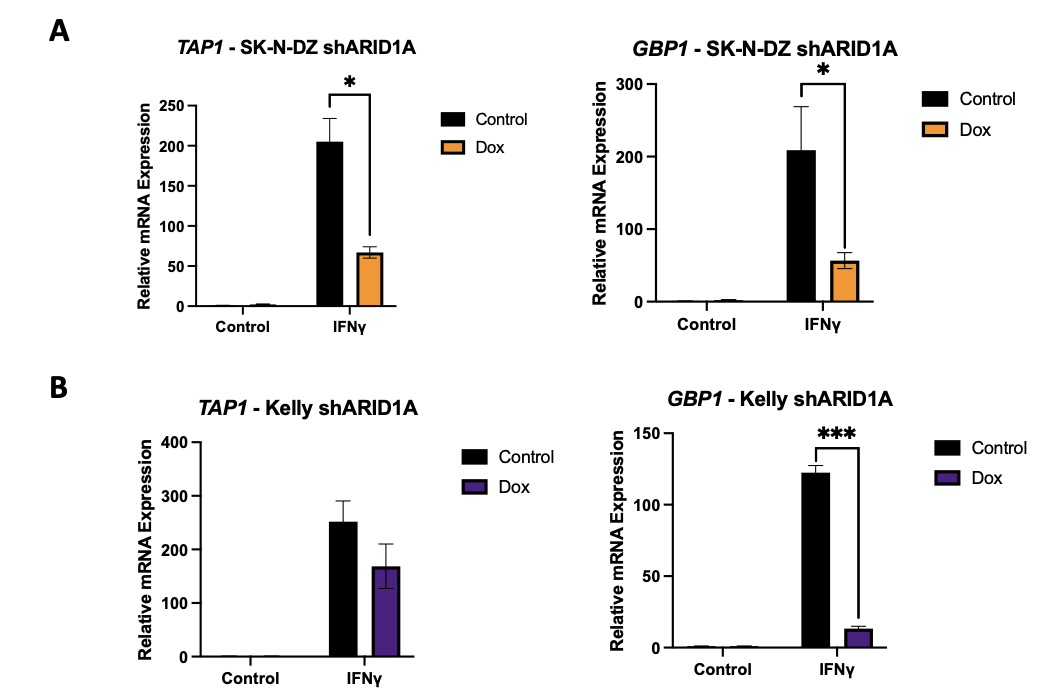


**Supplemental Figure 4: Knockdown of *ARID1A* leads to a decrease in the gene expression of immune related genes.**

**(A-B)** Inducible ARID1A knockdown NB clones were generated from **(A)** SK-N-DZ and **(B)** Kelly parental cells. Cells were treated with doxycycline for 4 days for ARID1A knockdown. Cells were treated with IFNγ for 24 hours prior to collection and subsequent analysis. Gene expression was measured by qPCR for antigen processing gene, *TAP1*, and another interferon responsive gene, *GBP1*. **(A-B)** *p < 0.1; **p < 0.01, ***p < 0.001, ****p < 0.0001.
